# Nutrients, Diet Quality, and Dietary Patterns in Patients with Inflammatory Bowel Disease: A Comparative Analysis

**DOI:** 10.3390/nu16183093

**Published:** 2024-09-13

**Authors:** Tingting Yin, Wenjing Tu, Yiting Li, Lina Huang, Yamei Bai, Guihua Xu

**Affiliations:** School of Nursing, Nanjing University of Chinese Medicine, Nanjing 210023, China; yori1998@163.com (T.Y.); 837017@njucm.edu.cn (W.T.); 20221074@njucm.edu.cn (Y.L.); huangln@njucm.edu.cn (L.H.)

**Keywords:** inflammatory bowel disease, dietary patterns, principal component analysis, diet quality

## Abstract

(1) Background: Diet plays an important role in the development of inflammatory bowel disease (IBD). There are a number of methods available to assess the diets of patients with IBD, including nutrients, dietary patterns, and various appraisal tools of diet quality. However, research on diet quality and dietary patterns in IBD populations is limited, and comparative evaluations of dietary intake in patients with IBD have not been performed. (2) Objectives: The aim of this study was to assess nutrients, the dietary patterns, and diet quality of patients with IBD and to investigate the relationship between dietary patterns, diet quality, and the adequacy of nutrient intake. (3) Methods: Three-day food records of 268 patients with ulcerative colitis (UC) and 126 patients with Crohn’s disease (CD) were collected to estimate nutrients and food groups, while dietary quality was assessed using the Dietary Inflammation Index (DII) and Mediterranean Diet Score (MDS). Dietary patterns were derived using principal component analysis (PCA). Participants’ nutrient intake, diet quality, and dietary patterns were compared. We used binary logistic regression to assess the relationship between dietary patterns (independent variable) and nutritional adequacy (dependent variable). (4) Results: In our sample, patients had inadequate energy, protein, and dietary fiber intake compared with Reference Nutrient Intake (RNI). Regarding micronutrients, intakes of potassium, zinc, selenium, vitamin A, vitamin C, vitamin E, sodium, calcium, iron, niacin, thiamin, and riboflavin were inadequate. Regarding food groups, the highest intakes were fruits, legumes, dairy products, and nuts. PCA revealed four dietary patterns, namely DP1, DP2, DP3, and DP4. Among UC patients, 96, 55, 69, and 48 patients adhered to DP1, DP2, DP3, and DP4 dietary patterns, respectively. Among CD patients, 41, 31, 34, and 20 patients complied with the dietary patterns of DP1, DP2, DP3, and DP4, respectively. There was no significant difference in dietary patterns between UC and CD patients. Compared with DP4 (high intake of mixed legumes and low intake of tubers), DP1 (high intake of cereals, tubers, vegetables and eggs) was more likely to ensure adequate intake of energy (OR, 2.96; 95% CI, 1.55, 5.62), protein (OR, 2.05; 95% CI, 1.06, 3.96), carbohydrates (OR, 3.55; 95% CI, 1.51, 6.59), thiamine (OR, 2.59; 95% CI, 1.36,4.93), niacin (OR, 2.75; 95% CI, 1.39, 5.42), phosphorus (OR, 2.04; 95% CI, 1.08, 3.85), zinc (OR, 2.43; 95% CI, 1.28, 4.63), and manganese (OR, 3.10; 95% CI, 1.60, 5.90), and DP2 (high intake of fruits, poultry, aquatic products, and nuts) was more likely to meet niacin requirements than DP4 (OR, 2.65; 95% CI, 1.28, 5.48). (5) Conclusion: This study clarifies our understanding of dietary intake, diet quality, and dietary patterns in adult patients with IBD. Future attention is needed to improve diet quality, emphasizing the importance of assessing and understanding patient dietary habits and increasing understanding of the factors that influence dietary intake in IBD in order to achieve optimal outcomes for patients with IBD.

## 1. Introduction

Ulcerative colitis (UC) and Crohn’s disease (CD) are collectively referred to as inflammatory bowel disease (IBD). Previous studies have shown that IBD is induced and maintained by environmental factors including diet, dysbiosis of the intestinal flora in genetically susceptible populations, and abnormal immune responses [1,2]. The exact interactions between these pathophysiologic factors are unknown. Nutrition plays a key role in the pathogenesis of IBD through its interactions with immunity, host barrier function, and gut flora [3].

Adequate nutritional intake depends on a balanced and nutritious diet. After diagnosis, it is particularly important for patients to meet the recommended goals for essential nutrients such as protein, fiber, calcium, and vitamins [4]. However, patients with IBD are at greater risk for protein-energy malnutrition and specific micronutrient deficiencies than the general population. Many studies [5,6,7] have investigated nutrient intake in IBD, and although their findings vary widely, in general, these studies reveal inadequate intakes of energy, fiber, vitamins C, D, B1, and B6, calcium,β-carotene, phosphorus, and magnesium. However, many of these studies had small sample sizes, e.g., only 54–126 IBD patients were recruited. The results of dietary surveys in IBD also vary considerably due to methodological heterogeneity, and dietary surveys do not take into account different geographical regions [8,9]. This is because the relative contribution of habitual dietary intake as well as genetic and environmental factors in the IBD phenotype may vary by region. However, this has not been specifically studied in patients from China.

Although nutrients and single food items are often of interest, it should be recognized that nutrients may act synergistically or antagonistically as part of a whole meal or daily dietary intake. Therefore, dietary patterns are considered to be of great clinical significance [10]. Principal Component Analysis (PCA) is a data-driven downscaling method for identifying such a posteriori dietary patterns, explaining much of the diversity of habitual intake among individuals in a given population [11,12]. In recent years, this method has attracted interest in the field of nutrition. An alternative approach to linking overall dietary intake to health outcomes is an a priori defined dietary score that is based on the assumption that foods are either harmful or beneficial and the scores’ adherence to target dietary recommendations. Here, we focus on two previously published dietary scores: the Dietary Inflammation Index (DII) [13] and the Mediterranean Diet Score (MDS) [14]. Using both dietary indices (a priori) and dietary patterns (a posteriori) to assess patients’ diets may provide a more insightful assessment. 

Only a few studies have addressed the relationship between dietary patterns and overall diet quality among IBD patients [15,16]. This information is critical because it is unclear whether diet quality improves with changes in dietary patterns in people with IBD, and which nutrients or a priori dietary scores may change. Given this evidence gap, the purpose of this study was to synthesize and characterize the usual dietary intake of adults with IBD and to investigate the relationship between dietary patterns, dietary quality, and nutrient intake adequacy. This information can be used to inform the development of clinical care resources, target interventions to improve the health of people with IBD, and design future trials of dietary interventions.

## 2. Materials and Methods

### 2.1. Study Design and Participant Selection

This exploratory, cross-sectional study collected 394 patients with IBD. The sample size was estimated using G*Power 3.1.9.7 with a significance level (α) of 0.05, a statistical power (1 − β) of 0.80, and a medium effect size (f^2^ = 0.15). The sample size required for logistic regression of the 10 predictors was estimated to be 187 individuals, and taking into account the 20% lost visit rate, 234 individuals should have been included in this study. IBD patients were recruited through posters and leaflets in IBD clinics and wards. 

Inclusion criteria were (a) ≥18 years of age; (b) meeting the diagnostic criteria of IBD in the Consensus Opinion on Diagnosis and Treatment of Inflammatory Bowel Disease (2018, Beijing) and confirmed by endoscopy and pathology [17,18]; (c) steroid-free clinical remission defined by The Crohn’s Disease Activity Index (CDAI) < 150 for CD patients or the Mayo score < 3 for UC patients for at least three months [17,18]; (d) having a smartphone and being able to use it proficiently. Patients were excluded if they had (a) > 1 bowel resection; (b) evidence of fistulizing or stricturing disease CD; (c) upper gastrointestinal disease; (d) an ostomy; (e) verbal communication disorders, or (f) inability to successfully complete meal recording.

Following informed consent and screening, demographic information was collected including age, sex, residence, education level, marital status, and type of IBD, together with basic anthropomorphic data (weight, height, body mass index).

### 2.2. Dietetic History and Nutritional Information 

Trained dietitians instructed all participants to use a three-day dietary diary to record in detail the names and estimated weights of all foods consumed over the three days (including two weekdays and one rest day to ensure proportional representation), and to use a standardized dietary intake toolkit to help quantify dietary intake. Participants were asked to record the names, brands, cooking methods, and portion sizes of all foods and beverages consumed. Portion sizes were recorded using measurements written on packaged food packages or cans, household measurements (e.g., spoons, cups), and photographs of foods in amorphous form (e.g., curries, stews). Mixed foods (e.g., dumplings, etc.) require the weight of the pasta to be recorded as well as the name and weight of the fillings; patients were advised to maintain their regular diets during the recording period, and no dietary changes were necessary. During the data collection process, the researchers provided patients with the 2016 edition of the Chinese Dietary Guidelines for Chinese Residents Food Standard Portion Reference Table to help patients assess specific food portion sizes [19]. Outpatient-recruited patients were contacted by researchers 2–3 days after enrollment to monitor and encourage compliance with food record completion, while inpatient patients were required to undergo three days of meal recording after resuming their daily meals. Immediately after returning the food records, the researchers checked the records for completeness and contacted participants for more detailed information when needed to improve the accuracy of the data. Nutrient intakes were expressed as units per day [d]. For nutrients, it was defined as meeting or exceeding the Reference Nutrient Intake (RNI) of the Dietary Guidelines for Chinese Residents.

Food records from the patients’ three days were entered into the web-based Healthy Gut [20], a software program that provides food information and occasional updates on composite foods based on the Dietary data that are entered by a researcher trained in the software. Patients’ average daily intake of nutrients was identified by calculating each participant’s average daily intake of energy, carbohydrates, protein, fat, dietary fiber, cholesterol, vitamin A, vitamin C, vitamin E, thiamin, riboflavin, niacin, calcium, phosphorus, iron, potassium, sodium, magnesium, zinc, selenium, copper, and manganese. In order to perform cluster analysis, food category information needed to be obtained. Ten food groups were identified from the three-day dietary diary data, including cereals, legumes, tubers, vegetables, fruits, poultry and meat, aquatic products, eggs, nuts, and dairy products (Table 1).

### 2.3. Dietary Inflammatory Index (DII) and the Mediterranean Diet Score (MDS)

The DII is a scoring system developed by Shivappa, based on a literature study that assessed the potential level of inflammation of dietary components [21]. The inflammatory effects of diet were assessed using 45 dietary parameters, including macronutrients, micronutrients, additional bioactive components, and ten whole foods. Individual intakes of each food parameter were normalized to global intakes using each parameter’s mean and standard deviation from 11 populations worldwide. The standardized intake scores (Z scores) were converted to proportions and centered. The centered proportions of these specific food intakes were multiplied by their inflammatory effect scores and summed to develop an overall DII score for the individual’s diet. An energy density approach was used, which calculated nutrient and final energy-adjusted DII scores per 1000 Kcal consumed. The participant’s DII score was the sum of each DII score. DII scores were higher for pro-inflammatory diets and lower for anti-inflammatory diets. In this study, 11/45 food parameters were available for DII calculations: energy, protein, total fat, iron, fiber, vitamin A, vitamin C, vitamin E, magnesium, zinc, and selenium.

The original 14-item MDS was developed in Spanish and later translated to English [22]. The English version was used to develop the self-administered version [23]. This version included pictures to facilitate comprehension of questions and simplified language. It is comprised of 13 items with yes/no options. The MDS includes four factors (Factor 1 reflects items related to meats and processed foods, Factor 2—olive oil and sauce items, Factor 3—fruits, vegetable, nuts (included tree nuts and ground nuts), and legumes, and Factor 4—fish and seafood) with a total of 13 questions with “yes” or “no” responses. The final score ranges from 0 to 13 (<5 = low adherence to MDP; >10 = high adherence to MDP). The self-administered version of the MDS is also available in Chinese languages [14].The Chinese version of the MDS scale was used in this study.

### 2.4. Statistical Analyses

Data analysis was performed using IBM SPSS Statistics for Windows Version 27.0 after the final participant’s data were collected. Categorical data were presented as numbers (percentages). Before statistical analysis, continuous data were explored for normality via visual inspection of histograms. Student’s *t*-test was used to analyze normally distributed continuous variables. Non-normally distributed continuous variables were expressed as medians [interquartile range (IQR)], and differences between groups were evaluated using the Mann–Whitney U test. 

Dietary patterns were derived from factor analysis and extracted by principal component analysis (PCA). Before applying PCA, the data’s suitability was assessed by conducting Bartlett’s test of sphericity and the Kaiser–Mayer–Olkin (KMO) test to ensure statistical correlation and sample size adequacy. A KMO value exceeding 0.5 is considered acceptable. Varimax rotation was used to simplify the structure and enhance interpretability. Factor retention for dietary patterns was determined by considering factors with a minimum eigenvalue of 1.0, the scree plot, and interpretability. The contribution of each food group to the pattern was measured through factor loadings. Factor loadings above 0.3 were considered significant [16]. Factor scores for each pattern represent the extent of alignment between the dietary intakes of the study subjects and the respective pattern. Higher factor scores indicate a stronger alignment. The distribution of individual adherence to each dietary pattern was re-categorized into four groups, ranging from quartile1 (Q1) representing the lowest quartile to quartile4 (Q4) representing the highest quartile. A higher score on a factor of dietary pattern indicated a stronger adherence to that particular dietary pattern. 

We categorized the adequacy of nutrient intake into dichotomous outcomes and used binary logistic regression to assess the relationship between dietary patterns (independent variable) and nutrient adequacy (dependent variable). In multivariate analyses, we adjusted for a variety of factors, including disease subtype, age, gender, place of residence, education, occupation, and body mass index. *p*-values ≤ 0.05 were considered statistically significant.

### 2.5. Ethics

This study was conducted following the Declaration of Helsinki and approved by approved by the Ethics Committee of the affiliated hospital (project approval number KY2022029). The study is registered in the Chinese Clinical Trials Registry (ChiCTR2200064943, accessed on 24 October 2022). Informed consent was obtained from all participants involved in the study.

## 3. Results

### 3.1. Demographic Characteristics of the Participants

A total of 394 participants were enrolled in this study. Among them, 251 were male (63.71%) and 143 were female (36.29%). More than half of the participants (68.02%) had UC, and 126 (31.98%) had CD. Most of the respondents (86.8%) lived in urban areas, the remaining (13.2%) lived in suburban or rural areas. The detailed demographic information and statistical description of the participants are presented in Table 2.

### 3.2. Nutrient Intake

Table 3 contains details of the participants’ nutritional intake. In our sample, patients had inadequate intake of energy, protein, and dietary fiber compared to RNI. Regarding micronutrients, potassium, zinc, selenium, vitamin A, vitamin C, vitamin E, sodium, calcium, iron, niacin, thiamine and riboflavin were under-consumed. In terms of food groups, the most inadequate intakes were fruits, legumes, dairy, and nuts.

### 3.3. Diet Quality

The Dietary Inflammatory Index (DII) ranged from −72 to 82 with a mean of −33.56 (median −43.89). The MDS ranges from 0 to 13. The findings of this study showed that the MDS ranged from 2 to 13 with a mean of 7.53 (median 7.50) in IBD patients. DII had a weaker but significant correlation with MDS (Spearman’s rho 0.118; *p* = 0.019) (Table 4).

### 3.4. Dietary Patterns

In our analysis, the KMO value was 0.594 (*p* < 0.001). Selecting components with factor loadings greater than 0.3, we identified four dietary patterns that accounted for 52.598% of the total variance, namely DP1, DP2, DP3, and DP4. DP1 was characterized by a high intake of grains, tubers, vegetables, and eggs. DP2 was characterized by a high intake of fruits, poultry and meat, aquatic products, and nuts. Meanwhile, DP3 is defined by a high intake of dairy products and a lower intake of eggs and nuts. Finally, DP4 is characterized by a high consumption of miscellaneous legumes and a lower intake of tubers. Among UC patients, 96, 55, 69, and 48 patients adhered to DP1, DP2, DP3, and DP4 dietary patterns, respectively. Among CD patients, 41, 31, 34, and 20 patients complied with the dietary patterns of DP1, DP2, DP3, and DP4, respectively. And there was no significant difference in dietary patterns between UC and CD (*X*^2^ = 1.176, *p* = 0.759) (Table 5).

Our findings showed significant associations between dietary patterns and energy, protein, carbohydrates, thiamin, niacin, phosphorus, and manganese (*p* < 0.05). To further explore the relationship between different dietary patterns, we chose DP4 as the reference population for OR. From the results, patients with DP1 were more likely to consume adequate energy, protein, carbohydrates, thiamine, niacin, phosphorus, and manganese than patients with DP4. This relationship persisted after adjusting for the confounders of energy, protein, carbohydrate, thiamine, niacin, phosphorus, and manganese (age, gender, region of residence, education, occupation, and BMI). DP2 was more likely to be associated with adequate niacin intake compared with patients with DP4. In addition, there was no significant difference in nutrient insufficiency between DP3 and DP4. As for DII and MDS, in this population, no statistically significant relationship was observed with any of the dietary patterns found (Table 6). 

## 4. Discussion

Several key findings of this study are novel in terms of nutrition and dietary intake in the Chinese IBD population. First, participants’ diet quality was generally suboptimal, with inadequate intake of energy, protein, fiber, calcium, iron, potassium, zinc, vitamin A, vitamin C, vitamin E, fruits, dairy products, legumes, and nuts and with excessive intake of carbohydrates and grains. Second, we observed low MD compliance in IBD patients in clinical remission, with DII scores indicating a dietary bias toward anti-inflammation, consistent with results obtained in other studies [3,24]. Finally, we also identified four different dietary patterns, and it is worth noting that we did not find any relationship between MDS or DII and dietary patterns. To the best of our knowledge, this is the first study to investigate the association between a posteriori dietary patterns and a priori dietary scores simultaneously in Chinese patients with IBD.

The inclusion of a larger sample of male subjects in this study is consistent with the characteristics of the Chinese population with IBD. A meta-analysis study of a Chinese cohort of patients with IBD (47 publications) by Li et al. [25] found a clear predominance of male patients with UC and CD. Our study found that the population with IBD was significantly more likely to be male than the female population. Adults with IBD consume insufficient energy, protein, and fiber during clinical remission compared with recommended requirements. Among them, female patients consumed less energy and protein and male patients consumed less dietary fiber. Low-fiber diets are often used by patients as a preventive measure. This can be explained by the fear of recurrent exacerbations of the disease [26]. A study by Cohen et al. [27] observed a reduced intake of fiber-rich foods, with deficiencies of up to 44% in this component. Intakes of important nutrients such as calcium, iron, potassium, zinc, and vitamins A, C, and E were also below recommended values in adult patients in remission with IBD. A review of micronutrients in IBD suggests that micronutrient deficiencies lead to further impaired immune responses in the gut, as well as inflammation due to increased production of reactive oxygen species [28]. Adequate calcium intake is critical since IBD is associated with an increased incidence of osteoporosis, in addition to pro-inflammatory cytokines that affect osteoblast activity during persistent inflammation. Studies by other researchers have also observed lower dietary calcium intake in patients with IBD [29,30]. These findings suggest that clinicians should take extra care to identify those with particularly poor intakes and implement dietary strategies to improve long-term intake of these nutrients and, in turn, improve diet quality. It is worth mentioning that the median intake of legumes and dairy products by the patients in our sample was 0. This result is quite different from the findings of dietary surveys in western populations [30]. The possible reason for this is that on one hand, the Western diet is based on proteins from meat, eggs and dairy, while the Chinese diet is based on starch from cereals. On the other hand, restrictive diets are more common in Chinese IBD patients [31], and legumes and dairy products are more likely to cause disease recurrence according to patients’ dietary beliefs. Another finding of this study is that people with IBD were identified as having inadequate intakes of major food groups considered central to a healthy diet, such as legumes, fruits, nuts, and dairy products, and excessive intakes of grains. Epidemiologic studies have shown that fruit intake is associated with a lower incidence of IBD [32]. Plant-based diets rich in dietary fiber appear to be beneficial to human health by promoting the development of diverse microbial ecosystems. It is important to note that elimination of lactose and dairy products, legumes, and fruits during IBD remission is not supported by any scientific evidence and does not follow the recommendations of the ESPEN IBD guidelines.

Diet quality is the degree of diversity of a dietary pattern or food group that defines a dietary pattern, relative to the recommendations included in the dietary guidelines. The DII and MDS are assessment tools used to quantify the quality of dietary intake by scoring food and/or nutrient intake. Using PCA, we identified four major dietary patterns, named DP1, DP2, DP3, and DP4. DP1 is characterized by high intake of grains, tubers, vegetables, and eggs; DP2 is characterized by high intake of fruits, poultry and meat, aquatic products, and nuts. DP3 is characterized by high intake of dairy products and low intake of eggs and nuts. DP4 is characterized by high intake of miscellaneous legumes and low intake of tubers. Most of the food groups in DP1 are in line with the recent dietary guidelines of the Asia Working Group and resemble a more “Mediterranean” dietary pattern. In recent years, the Mediterranean diet has been shown to have anti-inflammatory potential in chronic diseases such as IBD and to improve the diversity and abundance of gut microbiota and microbial metabolites [22]. After identifying dietary patterns, we began to examine the expected and unexpected relationships between diet quality and dietary composition and patterns. Our findings showed significant associations between dietary patterns and energy, protein, carbohydrates, thiamin, niacin, phosphorus, and manganese. DP1 was more likely to ensure adequate intake of energy, protein, carbohydrates, thiamine, niacin, phosphorus, and manganese compared to DP4. DP2 ensured a greater likelihood of meeting the recommended intake of niacin compared to DP4. No differences in nutrient intake were found between DP3 and DP4. Future studies should also consider multiple methods to capture dietary intake and may include the use of objective biomarkers.

In this cross-sectional study based on a Chinese IBD population, we observed a correlation between DII and MDS. The Western dietary pattern has typically been linked to higher levels of inflammatory markers in the body. By contrast, the Mediterranean diet is related to reduced inflammation levels [33]. However, surprisingly, we did not find a correlation between the dietary patterns we identified and MDS or DII. Today, the traditional Mediterranean diet is changing and becoming more westernized every day. Perhaps our participants primarily consumed a westernized Mediterranean diet rather than the traditional Mediterranean diet, which may explain why we did not find an association in our population [34]. Our study used 3-day food records to collect data, did not control for the same confounders as previous work, and had a different study population compared to the food frequency questionnaire.

There is a lack of data on nutritional intake of patients with IBD in China, as only a few studies have examined patients’ dietary patterns. Therefore, this study is a valuable addition to current literature and helps to improve our understanding of this particularly important population. This study included dietary intake information from 394 patients with IBD, and the use of a mobile app to collect prospective dietary intake data reduces recall bias and improves accuracy, making it the largest and most reliable assessment of dietary intake in IBD. In addition, this study is the first to objectively differentiate and compare associations between a posteriori dietary patterns and a priori dietary scores. Despite these strengths, the study had limitations. First, participants’ dietary records were collected over three days, limiting seasonal effects. Questionnaire-based dietary surveys may make the estimation of true nutrient intake more challenging. Vitamin D intake was not analyzed in this study when considering food composition, which may make generalization of the findings to other populations challenging. Additionally, in this study, food groups were defined based on the corresponding categories of the patient-recorded food charts, which sometimes resulted in combining potentially healthy and unhealthy foods in the same category. It should be noted that more detailed food grouping may be relevant for future studies. Finally, this study only measured micronutrient intake and not micronutrient status, which would be an important consideration in future studies.

## 5. Conclusions

In conclusion, we have identified preliminary associations between dietary components and nutrients and dietary patterns in patients with IBD in clinical remission. The lack of strong associations between DII and MDS and dietary patterns was unexpected. Further research is necessary to determine the underlying factors driving these patterns and how population-based interventions can improve the quality of nutrition in patients with gastrointestinal disease.

## Figures and Tables

**Table 1:** Groups and food items reported by study population.

Food Group	Detailed Food Items
Cereals	Cooked white rice, cooked rice with assorted mixtures, rice-noodle, non-fried noodles (white bread, steamed buns, noodles, dumplings)
Legumes	Soybeans, mung beans, red beans, soy milk, soy flour, tofu
Tubers	Sweet potato, potato, taro
Vegetables	Fresh legume vegetables, tomatoes, peppers, melon vegetables, green leafy vegetables, cabbage and other leafy vegetables, cruciferous vegetables
Fruit	Orange fruits, melon fruits, berry fruits, all other fresh fruits
Poultry and meat	Lean pork, fatty pork, beef, lamb, mutton, chicken, duck, goose, pigeon, quail
Aquatic products	Sea fish, freshwater fish, shrimp, crab
Eggs	Fresh egg, salted egg, preserved egg
Nuts	Peanuts, melon seeds, pumpkin seeds, watermelon seeds
Dairy	Liquid milk, milk powder, yogurt, cheese

**Table 2:** Descriptive data of the study participants (*N* = 394).

Participants’ Characteristics		All (%)	CD (%)	UC (%)
Gender	Male	251 (63.71%)	84 (66.67%)	167 (62.31%)
	Female	143 (36.29%)	42 (33.33%)	101 (37.69%)
Age (years)	18–40	273 (69.29%)	97 (76.98%)	176 (65.67%)
	41–59	99 (25.13%)	24 (19.05%)	75 (27.99%)
	≥60	22 (5.58%)	5 (3.97%)	17 (6.34%)
BMI Class (kg/m^2^)	Overweight (≥24)	71 (18.02%)	7 (55.56%)	54 (20.15%)
	Normal (18.5~23.9)	261 (66.24%)	99 (78.57%)	163 (60.82%)
	Underweight (<18.5)	62 (15.74%)	20 (15.87%)	51 (19.03%)
Education	Master’s degree or above	12 (3.05%)	8 (6.35%)	4 (1.49%)
	Junior college or bachelor	324 (82.23%)	102 (80.95%)	222 (82.84%)
	Secondary Education	52 (13.20%)	15 (11.90%)	37 (13.81%)
	Primary school or below	6 (1.52%)	1 (0.80%)	5 (1.86%)
Region	Urban	342 (86.80%)	113 (89.68%)	229 (85.45%)
	Suburban	22 (5.58%)	8 (6.35%)	14 (5.22%)
	Rural	30 (7.61%)	5 (3.97%)	25 (9.33%)
Occupation	Employee	280 (71.07%)	94 (74.60%)	186 (69.40%)
	Students	72 (18.27%)	18 (14.29%)	54 (20.15%)
	Unemployed	42 (10.66%)	14 (11.11%)	28 (10.45%)

Abbreviations: BMI = Body mass index; CD = Crohn’s disease; UC = ulcerative colitis

**Table 3:** Energy, macronutrient, micronutrient and food group intakes of IBD patients [M(P25,P75)].

Characteristics	Total Intake	Intake		RNI	
		Male	Female	Male	Female
**Macro nutrients**					
Energy, kcal	1402.57 (1324.45, 1480.69)	1261.83 (925.24, 1828.30)	1204.07 (710.88, 2058.81)	2100	1750
Protein, g	49.78 (46.51, 53.05)	40.00 (29.05, 59.87)	36.46 (24.24, 77.82)	65	55
Carbohydrate, g	259.30 (245.42, 273.18)	255.12 (169.78, 346.05)	235.07 (134.70, 361.78)	120	120
Dietary fiber, g	4.84 (4.43, 5.24)	3.35 (2.06, 5.92)	3.62 (2.09, 7.71)	25–30	25–30
Fat, g	20.15 (17.95, 22.36)	9.50 (4.80, 26.61)	11.28 (5.64,3 4.55)	20–30	20–30
**Micronutrients**					
Potassium, mg	980.35 (905.97, 1054.72)	707.30 (475.57, 1129.29)	714.47 (440.23, 1673.25)	2000	2000
Zinc, mg	6.34 (5.94, 6.75)	5.43 (3.84, 7.53)	4.66 (3.01, 9.87)	12.5	7.5
Selenium, μg	29.15 (26.77, 31.52)	21.68 (14.62, 37.27)	23.24 (12.08, 44.58)	60	60
Vitamin A, μg	198.33 (172.09, 224.57)	102.00 (42.50, 252.08)	126.40 (42.50, 308.14)	800	700
Vitamin C, mg	31.20 (27.25, 35.16)	10.67 (2.92, 34.14)	21.94 (5.06, 62.23)	100	100
Vitamin E, mg	6.61 (6.00, 7.23)	4.32 (3.17, 7.78)	4.32 (2.69, 9.52)	14	14
Sodium, mg	266.49 (234.74, 298.23)	125.63 (70.55, 321.41)	141.57 (75.39, 428.27)	1500	1500
Calcium, mg	195.97 (177.38, 214.56)	101.04 (68.55, 222.09)	149.87 (73.15, 340.86)	800	800
Iron, mg	10.34 (9.58, 11.10)	8.27 (5.00, 13.79)	7.71 (4.21, 15.85)	12	20
Phosphorus, μg	664.08 (623.31, 704.85)	557.95 (408.56, 819.10)	509.50 (346.25, 1047.42)	600	600
Magnesium, mg	199.01 (186.49, 211.52)	166.37 (123.17, 244.27)	154.73 (92.70, 316.20)	280	280
Copper, mg	1.31 (1.04, 1.57)	0.87 (0.63, 1.38)	0.84 (0.50, 1.68)	0.8	0.8
Manganese, mg	4.24 (3.89, 4.60)	3.50 (2.33, 5.05)	3.08 (1.74, 5.98)	4.5	4.5
Niacin, mg	9.20 (8.57, 9.83)	7.36 (5.05, 12.07)	6.66 (4.27, 13.31)	15	12
Cholesterol, mg	221.10 (193.93, 248.27)	108.00 (18.10, 286.00)	129.50 (25.80, 374.20)	<300	<300
Thiamin, mg	0.85 (0.76, 0.93)	0.71 (0.53, 1.07)	0.71 (0.42, 1.15)	1.4	1.2
Riboflavin, mg	0.52 (0.46, 0.57)	0.37 (0.29, 0.56)	0.37 (0.26, 0.77)	1.4	1.2
**Food groups**					
Cereals, g	456.67 (340.00, 560.00)	473.33 (351.67, 577.14)	435.56 (330.11, 520.00)	50–150	50–150
Legumes, g	0.75 (0.00, 6.50)	0.00 (0.00, 6.67)	0.00 (0.00, 6.67)	50–150	50–150
Tubers, g	24.86 (0.00, 51.42)	22.08 (0.00, 50.00)	30.00 (0.00, 50.00)	50–100	50–100
Vegetables, g	207.00 (0.92, 294.33)	208.33 (115.00, 293.67)	203.33 (138.33, 300.00)	300–500	300–500
Fruit, g	26.06 (0.92, 79.67)	20.00 (0.00, 66.67)	36.67 (0.00, 90.00)	200–350	200–350
Poultry, g	122.25 (73.97, 176.89)	126.67 (75.84, 186.67)	116.67 (67.78, 157.78)	40–75	40–75
Aquatic products, g	34.23 (2.67, 70.66)	33.33 (0.00, 75.50)	40.00 (5.00, 65.00)	40–75	40–75
Eggs, g	53.75 (31.74, 74.33)	56.67 (33.33, 77.34)	50.00 (26.67, 66.67)	40–50	40–50
Nuts, g	16.95 (1.01, 50.95)	16.67 (0.00, 50.00)	21.67 (0.00, 56.25)	25–35	25–35
Dairy, g	1.75 (0.00, 34.69)	0 (0.00, 20.00)	0 (0.00, 50.00)	300	300

**Table 4:** Dietary Inflammatory Index and Mediterranean Diet Score of IBD patients.

Score Categories	Mean	Median	SD	Minimum	Maximum	Percentiles			*p*-Value
						25th	50th	75th	
**Diet Quality Indices**									0.019
Dietary Inflammation Index	−33.56	−43.89	29.33	−72	82	−53.24	−43.89	−19.53	
The Mediterranean Diet Score	7.53	7.50	2.64	2	13	6.00	7.50	9.00	

**Table 5:** Dietary patterns identified in the study sample through principal component analysis.

Food Group	Dietary Pattern			
	DP1 (UC = 96, CD = 41)	DP2 (UC = 55, CD = 31)	DP3 (UC = 69, CD = 34)	DP4 (UC = 48, CD = 20)
Cereals	**0.721**	−0.007	0.123	−0.053
Legumes	0.085	−0.079	0.096	**0.872**
Tubers	**0.425**	0.104	0.060	**−0.330**
Fresh vegetables	**0.676**	0.210	−0.107	0.299
Fresh fruit	−0.126	**0.763**	0.088	0.052
Poultry	0.214	**0.579**	0.024	−0.203
Aquatic products	0.237	**0.305**	0.033	−0.053
Eggs	**0.541**	−0.017	**−0.408**	0.088
Nuts	0.017	**0.539**	**−0.601**	0.140
Dairy	0.056	0.252	**0.781**	0.179

Variables bolded have an Eigenvalue > 0.3.

**Table 6:** Relationship between dietary patterns and nutrients, Dietary Inflammatory Index (DII), Mediterranean Diet Score (MDS).

Predictor	Dietary Pattern	*p*	OR	95% CI		*p*	aOR *	95% CI	
				Lower	Upper			Lower	Upper
Energy	1	<**0.001**	**2.96**	**1.55**	**5.62**	<**0.001**	**3.02**	**1.57**	**5.81**
	2	0.11	1.78	0.88	3.59	0.11	1.78	0.87	3.62
	3	0.11	1.75	0.89	3.46	0.10	1.78	0.89	3.49
Protein	1	**0.03**	**2.05**	**1.06**	**3.96**	**0.03**	**2.17**	**1.10**	**4.25**
	2	0.17	1.65	0.81	3.39	0.13	1.76	0.85	3.64
	3	0.15	1.67	0.84	3.35	0.13	1.71	0.85	3.46
Fat	1	0.44	0.78	0.42	1.45	0.56	0.83	0.44	1.56
	2	0.27	1.45	0.75	2.79	0.23	1.51	0.77	2.96
	3	0.86	0.94	0.50	1.80	0.86	0.94	0.49	1.82
Carbohydrate	1	<**0.001**	**3.55**	**1.91**	**6.59**	<**0.001**	**3.56**	**1.90**	**6.68**
	2	0.33	1.39	0.71	2.73	0.37	1.37	0.69	2.72
	3	0.09	1.74	0.91	3.31	0.11	1.71	0.89	3.28
Dietary fiber	1	0.97	0.99	0.54	1.81	0.94	1.03	0.56	1.89
	2	0.59	0.83	0.43	1.62	0.66	0.86	0.44	1.69
	3	0.52	0.81	0.43	1.54	0.50	0.80	0.42	1.53
Cholesterol	1	0.73	0.90	0.49	1.66	0.97	0.99	0.52	1.86
	2	0.38	1.34	0.69	2.60	0.31	1.43	0.72	2.82
	3	0.88	1.05	0.55	2.00	0.88	1.05	0.55	2.03
Thiamine	1	<**0.001**	**2.59**	**1.36**	**4.93**	<**0.001**	**2.66**	**1.38**	**5.12**
	2	0.14	1.69	0.84	3.42	0.14	1.72	0.84	3.51
	3	0.13	1.68	0.85	3.32	0.13	1.69	0.85	3.34
Riboflavin	1	0.77	1.10	0.58	2.10	0.56	1.22	0.63	2.36
	2	0.58	1.22	0.61	2.45	0.45	1.32	0.65	2.70
	3	0.71	1.14	0.58	2.24	0.73	1.13	0.57	2.25
Niacin	1	<**0.001**	**2.75**	**1.39**	**5.42**	<**0.001**	**2.92**	**1.45**	**5.85**
	2	**0.01**	**2.65**	**1.28**	**5.48**	**0.01**	**2.76**	**1.31**	**5.81**
	3	0.06	2.02	0.99	4.12	**0.05**	**2.06**	**1.00**	**4.26**
Vitamin A	1	0.48	1.25	0.67	2.35	0.28	1.43	0.75	2.74
	2	0.79	1.10	0.55	2.20	0.62	1.20	0.59	2.43
	3	0.92	1.03	0.53	2.02	0.92	1.04	0.52	2.05
Vitamin C	1	0.62	1.17	0.62	2.21	0.35	1.37	0.71	2.63
	2	0.31	1.42	0.72	2.81	0.20	1.59	0.79	3.20
	3	0.82	1.08	0.56	2.11	0.76	0.79	0.56	2.19
Vitamin E	1	0.75	1.10	0.60	2.05	0.71	1.13	0.60	2.12
	2	0.90	0.96	0.48	1.89	0.95	0.98	0.49	1.95
	3	0.96	0.94	0.49	1.82	0.88	0.95	0.49	1.84
Calcium	1	0.81	0.93	0.50	1.72	0.90	1.04	0.55	1.96
	2	0.77	1.10	0.57	2.15	0.50	1.26	0.64	2.51
	3	0.88	1.05	0.55	2.00	0.81	1.08	0.56	2.09
Phosphorus	1	**0.03**	**2.04**	**1.08**	**3.85**	**0.04**	**2.00**	**1.05**	**3.81**
	2	0.41	1.34	0.66	2.71	0.37	1.38	0.68	2.81
	3	0.20	1.56	0.80	3.05	0.20	1.56	0.79	3.07
Potassium	1	0.61	1.18	0.62	2.24	0.49	1.26	0.65	2.43
	2	0.64	1.18	0.59	2.37	0.57	1.23	0.61	2.51
	3	0.57	1.22	0.62	2.38	0.54	1.24	0.63	2.44
Sodium	1	0.57	0.84	0.45	1.56	0.89	0.95	0.50	1.81
	2	0.56	1.22	0.63	2.37	0.46	1.29	0.65	2.57
	3	0.61	0.84	0.44	1.62	0.60	0.84	0.43	1.64
Magnesium	1	0.09	1.69	0.92	3.13	0.11	1.68	0.90	3.13
	2	0.95	1.02	0.52	2.04	0.92	1.04	0.52	2.08
	3	0.42	1.31	0.68	2.51	0.41	1.32	0.68	2.54
Iron	1	0.12	1.62	0.88	2.96	0.14	1.59	0.86	2.93
	2	0.75	0.90	0.45	1.76	0.80	0.91	0.46	1.81
	3	0.78	1.10	0.58	2.09	0.80	1.09	0.57	2.07
Zinc	1	**0.01**	**2.43**	**1.28**	**4.63**	**0.01**	**2.38**	**1.24**	**4.56**
	2	0.09	1.83	0.91	3.69	0.09	1.85	0.91	3.74
	3	0.35	1.39	0.70	2.76	0.35	1.39	0.70	2.77
Selenium	1	0.15	1.60	0.84	3.03	0.12	1.68	0.87	3.24
	2	0.26	1.49	0.74	2.99	0.27	1.49	0.73	3.05
	3	0.32	2.07	1.07	4.03	0.31	2.10	1.07	4.13
Copper	1	0.17	1.58	0.83	3.04	0.11	1.73	0.89	3.39
	2	0.69	1.16	0.56	2.39	0.67	1.17	0.56	2.47
	3	0.37	1.37	0.69	2.73	0.35	1.40	0.70	2.82
Manganese	1	<**0.001**	**3.1**	**1.6**	**5.9**	<**0.001**	**3.02**	**1.57**	**5.80**
	2	0.09	1.1	0.5	2.3	0.79	1.10	0.53	2.30
	3	0.75	1.1	0.6	2.3	0.77	1.11	0.55	2.24
MDS	1	0.92	0.9	0.3	2.7	0.81	1.14	0.39	3.39
	2	0.64	1.3	0.4	4.4	0.42	1.68	0.48	5.89
	3	0.06	0.4	0.1	1.0	0.07	0.37	0.13	1.06
DII	1	0.49	0.8	0.4	1.5	0.44	0.78	0.41	1.46
	2	0.93	1.0	0.5	2.1	0.99	1.00	0.50	2.01
	3	0.77	0.9	0.5	1.8	0.76	0.90	0.46	1.75

DP4 is used as the reference comparator for estimations of OR, OR: odds ratio, aOR: adjusted odds ratio, * adjusted for age, gender, BMI, education, occupation, area of residence, and IBD type; values bolded have *p* < 0.05.

## Data Availability

The original contributions presented in the study are included in the article; further inquiries can be directed to the corresponding author.
